# How does self-investment influence intimate relationships? Examining the mediating role of the self-other link

**DOI:** 10.3389/fpsyg.2026.1741886

**Published:** 2026-09-18

**Authors:** Xiangli Guan, Yaqi Zhang, Jingjing Wang, Xuejiao Li, Sha Shen, Yue Yuan, Mary C. Jobe, Md Zahir Ahmed

**Affiliations:** 1 School of Teacher Education, Yuxi Normal University, Yuxi, China; 2 College of Educational Science and Technology, Northwest Minzu University, Lanzhou, China; 3 School of Education, Yunnan Minzu University, Kunming, China; 4 Department of Psychological and Brain Sciences, The George Washington University, Washington, DC, United States; 5 School of Psychology, Zhejiang Normal University, Jinhua, China

**Keywords:** intimate relationships, self-inclusion of others, self-investment, self-other link, structural equation modeling

## Abstract

**Introduction:**

This cross-sectional study explored the psychological pathways underlying the establishment of intimate relationships based on Self-Determination Theory and Egocentric Categorization Theory, with a focus on the core role of self-investment and examining the chain mediating effect of self-inclusion of others and the self-other link.

**Methods:**

A sample of 369 Chinese students was recruited using convenience sampling technique. Common method variance (CMV) was tested using Harman’s single-factor test and Exploratory Factor Analysis (EFA). A regression-based mediation analysis using Hayes’ PROCESS macro was applied to analyze the relationships among self-investment, self-inclusion of others, the self-other link, and intimate relationships.

**Results:**

Significant positive correlations were found among self-investment, self-inclusion of others, the self-other link, and intimate relationships (*p* < 0.01). A mediation analysis revealed three indirect paths through which self-investment was associated with intimate relationships: self-investment → self-inclusion of others → intimate relationships (31.82%), self-investment → self-other link → intimate relationships (18%), and self-investment → self-inclusion of others → self-other link → intimate relationships (27.27%).

**Discussion:**

This study reveals the internal mechanism by which self-investment influences the establishment of intimate relationships through a two-stage chain path of “cognitive inclusion-emotional connection,” providing theoretical basis and practical significance among Chinese students.

## 1 Introduction

All human behavior and experiences can originate from relationships (Greenberg and Greenberg, 2019). Liang (2018) proposed that individuals need to handle three types of relationships—between humans and things, between other humans, and between humans and their inner selves—which shape experiences and promote psychological maturity (Liang, 2018). The object relations theory holds that the human mind is a “relational being,” and psychological composition relies on relationship internalization (Che and Guo, 2018); the quality of such relationships affects mental health (Finkel et al., 2017). In China’s collectivist culture, Fei (1998) used the concept of “chaxu geju” (the differential mode of association) to summarize the characteristics of interpersonal relationships, noting that humans are “individuals in relationships” (Fei, 1998), and positive interpersonal relationships are the cornerstone of social harmony (Finkel et al., 2017). However, in reality, college students tend to fall into relationship dilemmas (e.g., egocentrism) due to their psychological traits, pressures, and insufficient capabilities, leading to loneliness and alienation (Ren et al., 2020). This study aims to explore the psychological pathways for establishing intimate relationships and verify the importance of mutual respect, understanding, and two-way giving.

### 1.1 Self-investment, self-inclusion of others, self-other link, and intimate relationships

Self-investment is resources (time, emotion, energy) individuals devote to relationships (Gerbasi and Prentice, 2013; Harandi, 2021). Self-inclusion of others (IOS) is how much one integrates others into their self-concept (Aron et al., 1991, 1992). The self-other link is the closeness one feels with others. Intimate relationships are deep, long emotional bonds (romantic, friendship, and/or familial) (Felix et al., 2021; Shen, 2025). In them, self-investment may affect relationship quality or stability by shaping one’s perception of and interaction with partners (Slotter and Gardner, 2009). IOS, a cognitive structure, reflects incorporating partners’ traits, resources and perspectives into the self-concept. The self-other link broadly refers to interaction patterns and emotional connections between individuals. Although both the IOS and self-other link are closely related, they are theoretically distinct. IOS highlights the cognitive aspect of relationship development, and the self-other link incorporates mostly partner’s traits and perspectives. Moreover, IOS highlights cognitive integration that influences individual perceptions and behaviors. In contrast, the self-other link emphasizes the emotional bond and relational closeness one can have, shaping the intimacy and satisfaction of any relationship (Branand et al., 2019).

#### 1.1.1 Self-investment and self-inclusion of others

Self-investment may boost the self-inclusion of others. When individuals focus more on self-development and have higher self-esteem and self-efficacy, they tend to view themselves and their partners positively; easily integrating partners into their self-concept. This process works in three ways, first by enhancing self-esteem and security. This improves self-value perception, reducing partner dependence and insecurity; those with stronger secure relationships build intimate, trusting bonds and see partners as integral with the self (Robinson and Cameron, 2012). Second, aiding positive self-presentation enables individuals to confidently show strengths to partners, increasing partners’ affection and recognition, and fostering healthy interactions (Øverup et al., 2015). Third, increasing shared activities drives active participation in the relationship, deepening mutual understanding and connection (Quintard et al., 2020).

#### 1.1.2 Self-inclusion of others and intimate relationships

Self-inclusion of others is closely linked to intimate relationship quality. Studies show that higher self-inclusion correlates with greater relationship satisfaction, commitment, and intimacy, due to three effects. The first being it strengthens mutual empathy—integrating partners into one’s self-concept as well as helping one think from the other’s perspective to understand needs and reduce conflicts (Gordon and Chen, 2013). The second is that it promotes resource sharing, making individuals see partners’ resources as their own, boosting willingness to help and cope with challenges (Aron et al., 1991). Third, this effect has shown to increase relationship investment, leading to a greater focus on the shared bond and willingness to spend time/energy with one another, overall enhancing relationship stability (Weiser and Weigel, 2016).

#### 1.1.3 Self-investment, self-other link, and intimate relationships

Self-investment affects intimate relationships directly and indirectly via the self-other link, such as through, fostering equal, supportive interactions or shaping power distribution/decision-making. Studies show linking relationships to self-expansion boosts satisfaction and involves integrating others into the self (Montoya et al., 2018). The time it takes to establish intimate relationships depends on activities and shared information. One study found strangers formed intimacy in 45 min, with one pair later marrying (Aron et al., 1997). Shared intimate knowledge is a form of self-investment. A meta-analysis (Montoya et al., 2018) noted attraction-related behaviors (eye contact, mimicry) tie to trust/relationship development, with direct acts (proximity and conversation) more linked to attraction than indirect ones, as they involve greater self-investment. Interaction quality also matters; direct contact and in-depth communication build strong emotional and cognitive integration quickly, fostering resonance. Self-investment (emotional, cognitive, and physical openness) boosts closeness, such as through emotional depth and vulnerability. While direct acts aid initial intimacy, indirect acts (support, listening) sustain it later. Ideal intimate relationship development needs multiple interaction methods, combining direct vitality and indirect patience.

### 1.2 Hypotheses of the study

Therefore, we argue that self-investment may predict intimate relationships through the mediating roles of self-inclusion of others and the self-other link. The theoretical hypothesis model of this study is presented in Figure 1:

**Figure 1:**
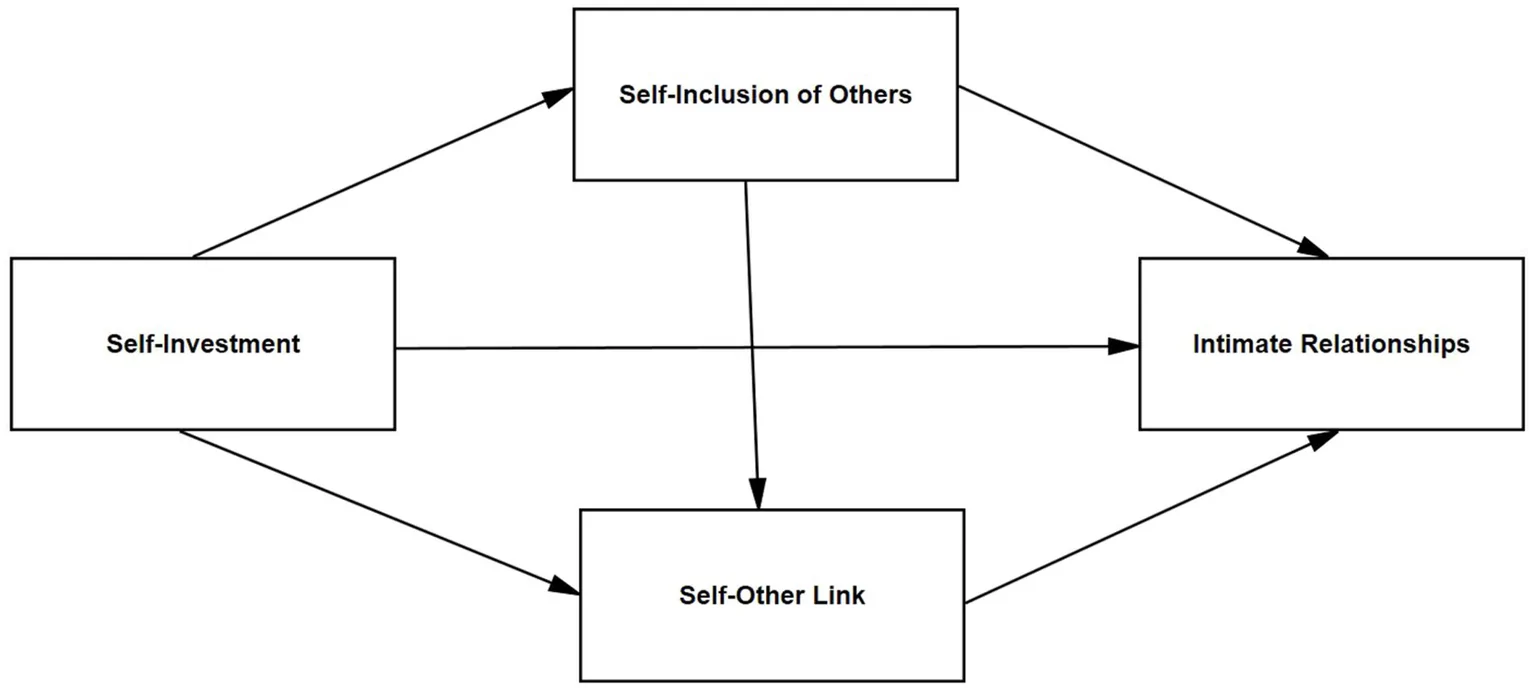
Hypothetical model.

*Hypothesis 1*: Self-investment can directly predict intimate relationships.

*Hypothesis 2*: Self-investment can indirectly predict intimate relationships through self-inclusion of others.

*Hypothesis 3*: Self-investment can indirectly predict intimate relationships through the self-other link.

*Hypothesis 4*: Self-investment can indirectly predict intimate relationships through the chain mediating role of self-inclusion of others and the self-other link.

## 2 Materials and methods

### 2.1 Participants

A convenience sampling method was adopted to select 450 undergraduate students from 8 provinces (Anhui, Beijing, Gansu, Hubei, Shandong, Sichuan, Yunnan, and Zhejiang) of China for this study as the participants. According to the power analysis results using G*Power, with the effect size *f* set at 0.15, the significance level *α* at 0.05, and the statistical power at 80%, the total sample size required for this study was calculated to be 351 participants. After the survey, the completed scales were re-examined, and 31 invalid questionnaires were excluded—including those with missing responses and those that failed the attention check questions. Finally, a total of 369 valid questionnaires were obtained. The participants were aged between 18 and 21 years (*M* = 19.3; *SD* = 0.74). Among them, 274 participants (74.3%) were female, and 95 participants (25.7%) were male.

### 2.2 Measures

#### 2.2.1 Self-investment

This study adopted a self-investment questionnaire adapted from the version developed by Brown et al. (2014). The scale consists of 5 items, with responses scored on a 1 (*strongly disagree*) to 7 (*strongly agree*) Likert scale. It assesses self-investment from two aspects: the specific content of investment (e.g., time, emotion devoted to the relationship) and the overall level of investment. A higher total score indicates that the individual has a stronger tendency to engage in self-investment in interpersonal relationships. This questionnaire has been widely validated and applied in previous studies (Brown et al., 2014; Kirk et al., 2015; Guan et al., 2023). In the current study, the internal consistency reliability (Cronbach’s α coefficient) of this scale was 0.98, indicating excellent reliability.

#### 2.2.2 Self-inclusion of others

This study uses the Inclusion of Others in the Self (IOS) scale (Aron et al., 1997) to assess how individuals integrate partners into their self-concept. The widely used scale, simple with strong psychometrics (Aron et al., 2022), adopts a visual approach—7 pairs of overlapping “self-other” circles (no verbal responses). More overlap means closer integration, less means clearer boundaries; participants pick the pair matching their intimate relationship. It has proven validity: it distinguishes intimacy levels in American college students and correlates positively with relationship quality (Aron et al., 1992, 2022). Its intuitive graphics also aid cross-cultural use by transcending linguistic/cultural barriers (Aron et al., 2022).

#### 2.2.3 Self-regulation

The self-other link scale in this study was adapted from the “Possession-Self Link Scale” originally developed by Ferraro et al. (2011). The key adjustment was changing the measurement target from “possessions” (e.g., personal items) to “others” (i.e., intimate partners), to fit the research focus on interpersonal relationships. The adapted scale includes 7 items, covering four core dimensions: perceived connection between the self and others, the importance of others to the self, positive emotions elicited by interactions with others, and negative emotions triggered by others’ misfortunes or harm. Responses were scored on a 1 to 7 Likert scale, with higher scores indicating a closer and more intense self-other link. The adapted Self-Other Link Scale has been validated in previous research (Guan, 2025). This adapted version has also been used in multiple studies (Ferraro et al., 2011; Stander and Coxen, 2017) to ensure its validity. The confirmatory factor analysis in this study yielded acceptable results: χ2/*df* = 8.02, CFI = 0.95, NFI = 0.95, GFI = 0.91, RMR = 0.09. In the present study, the Cronbach’s *α* coefficient of this scale was 0.96, demonstrating high internal consistency.

#### 2.2.4 Intimate relationships

To measure the quality of intimate relationships, this study used the Chinese translated version of the shortened Quality of Relationship Index (QRI), which was initially revised by Patrick et al. (2007) and later translated into Chinese by Zhou (2024). This scale aims to assess individuals’ subjective perception and evaluation of their current intimate relationships (e.g., satisfaction with the relationship, sense of closeness). It contains 6 items, with each item scored on a 1 to 7 Likert scale. A higher total score indicates that the individual has a higher level of subjective satisfaction with their current intimate relationship. Prior research has verified the psychometric properties of this scale: its internal consistency reliability (Cronbach’s α) reached 0.91, and its criterion validity ranged from 0.40 to 0.70 (Zhou, 2024), confirming its good reliability and validity. In the current study, the Cronbach’s α coefficient of this scale was 0.95, indicating that it is suitable for the sample of this study.

### 2.3 Procedure

Two survey modes were implemented. On-site anonymous survey: Psychology teachers administered on-site anonymous tests in classrooms. Online survey: Trained homeroom teachers distributed questionnaires to the WeChat groups of each class for online data collection. After collecting all questionnaires, the returned ones were verified, and 31 invalid questionnaires were eliminated. Ultimately, 369 valid questionnaires were obtained, with an effective response rate of 82%. Software including SPSS 26.0 and Process v.3.3 was used for data management and data analysis.

### 2.4 Statistical analysis

We utilized SPSS 26.0 for initial data management, including quality checks and exploratory assessment of distributional properties. After preparing the dataset, we conducted descriptive statistics and basic inferential tests within SPSS. To evaluate indirect (mediation) effects, however, we turned to a more specialized toolkit. Accordingly, all mediation analyses were executed using Hayes’ PROCESS macro (v. 3.3) (Hayes, 2018), a regression-based mediation approach employing employed a robust bootstrapping method with 5,000 iterations to generate confidence intervals. The threshold for statistical significance was set at *p* < 0.05.

### 2.5 Ethical approval

This study involved human participants and strictly adhered to the ethical principles outlined in the Helsinki Declaration and its subsequent amendments. The research protocol was approved by the Ethics Committee of Yuxi Normal University, Yuxi, China (Approval No. 2024019; Date: 11 October 2024). Prior to the survey, all participants were informed of the research objectives, potential costs and benefits, estimated time required, and data confidentiality measures. After fully understanding the nature of the study, all participants provided written informed consent.

## 3 Results

### 3.1 Common method variance

This study adopted self-reported scales, which may lead to common method variance (CMV). Therefore, procedural designs were implemented prior to the survey to mitigate this issue. The first was anonymity assurance, where respondents were explicitly assured that their answers would remain completely anonymous and that the data would only be used for academic research. This measure helps reduce their social desirability bias, encouraging them to answer truthfully rather than providing “correct” or “appropriate” responses. Second, psychological separation was used; in the questionnaire design, items of key variables (e.g., independent variables and dependent variables) were randomized and not placed together explicitly. Additionally, other scales or screening questions were inserted between them to prevent respondents from easily inferring the research hypotheses, thereby reducing their tendency to deliberately maintain consistency in answers. Third, diversified scale formats, where different scale anchors (e.g., a 7-point Likert scale for one variable and a progressive graphic scale for another), different directions (some items scored positively and others negatively), and different item expression styles were used for independent and dependent variables.

After data collection, statistical methods were employed to test the severity of CMV and control it, if necessary. In this study, Harman’s single-factor test was used to examine CMV in the data processing procedure. Although Harman’s single-factor test was conducted, it is noteworthy that this particular method often limits the sensitivity for detecting all forms of CMV, mostly the subtle biases in self-report data. Exploratory Factor Analysis (EFA) was also conducted on all variable items, and the variance proportion explained by the first unrotated factor was checked. The results showed that a total of 2 factors with eigenvalues greater than 1 explained 58.91% of the variance, and the variance explained by the first factor was 32.79%, which was lower than the critical threshold of 40%. This indicates that the impact of common method variance was minimal and did not signify any major concern regarding CMV.

### 3.2 Descriptive statistics analysis

Table 1 presents the descriptive statistical results of all research variables. According to the criteria proposed by Kim (2013), data is judged to have a non-normal distribution if the absolute value of skewness is greater than or equal to 2 and the absolute value of kurtosis is greater than or equal to 7. In this study, the skewness coefficients of all variables ranged from −0.13 to 0.66, and the kurtosis coefficients ranged from −0.95 to 0.94. All values were below the aforementioned critical thresholds, indicating that the data conformed to a normal distribution. In addition, pairwise correlations among self-investment, self-inclusion of others, the self-other link, and intimate relationships were all significantly positive, and all correlation results reached the statistical significance level (*p* < 0.01).

**Table 1:** Descriptive statistics of the study variables (*n* = 369).

Variables	*M*	*SD*	Skewness	Kurtosis	1	2	3	4
1. Self-investment	4.13	0.05	0.61	0.94	1			
2. Self-inclusion of others	3.60	0.08	0.66	−0.42	0.374**	1		
3. Self-other link	4.40	0.06	−0.10	−0.95	0.401**	0.723**	1	
4. Intimate relationships	4.16	0.06	−0.13	−0.86	0.402**	0.630**	0.660**	1

M, mean; SD, standard deviation. *p < 0.05; **p < 0.01; ***p < 0.001.

### 3.3 Analysis of predictive relationships among self-investment, self-inclusion of others, self-other link, and intimate relationships

To analyze the impact of self-investment on intimate relationships and the roles of self-inclusion of others and self-other link in this process, the SPSS macro PROCESS program was used to test the mediating effects. In the analysis, the skewness-corrected percentile Bootstrap method was adopted to test mediating effects; a Bootstrap 95% confidence interval (CI) that does not include 0 indicates a significant mediating effect. The test of the mediation model requires estimating the parameters of two regression equations: first, the direct effect of the independent variable (self-investment) on the dependent variable (intimate relationships); second, the mediating effect (indirect effect) of the mediating variables (self-inclusion of others, self-other link) in the relationship between the independent variable (self-investment) and the dependent variable (intimate relationships).

A mediating effect exists if the model meets the following conditions: (1) The predictive effect of self-investment on intimate relationships is significant; (2) The predictive effects of self-investment on self-inclusion of others and self-other link are significant; (3) The predictive effects of self-inclusion of others and self-other link on intimate relationships are significant; (4) The predictive effect of self-inclusion of others on the self-other link is significant. The results of the regression analysis are shown in Table 2. Self-investment, self-inclusion of others, and the self-other link can all independently predict intimate relationships. The model results are consistent with expectations. Specifically, self-investment positively predicts self-inclusion of others (*β* = 0.60, *p* < 0.001); self-investment positively predicts the self-other link (*β* = 0.15, *p* < 0.001); self-investment positively predicts intimate relationships (*β* = 0.1, *p* < 0.05); self-inclusion of others positively predicts the self-other link (*β* = 0.39, *p* < 0.001); self-inclusion of others positively predicts intimate relationships (*β* = 0.23, *p* < 0.001); and the self-other link positively predicts intimate relationships (*β* = 0.52, *p* < 0.001). Therefore, there exists a chain mediating effect of the self-inclusion of others and self-other link in the impact of self-investment on intimate relationships (see Figure 2).

**Table 2:** Intermediary effect model test (*n* = 369).

Variable	Model 1			Model 2			Model 3		
	Self-inclusion of others			Self-other link			Intimate relationships		
	*β*	*RE*	*t*	*β*	*RE*	*t*	*β*	*RE*	*t*
Self-investment	0.60	0.71	8.40***	0.15	0.05	3.30***	0.1	0.04	2.48*
Self-inclusion of others				0.39	0.03	13.09**	0.23	0.03	7.15***
Self-other link							0.52	0.05	11.33***
*R^2^*	0.16			0.41			0.6		
*F*	70.62***			129.30***			181.17***		

*p < 0.05; **p < 0.01; ***p < 0.001.

**Figure 2:**
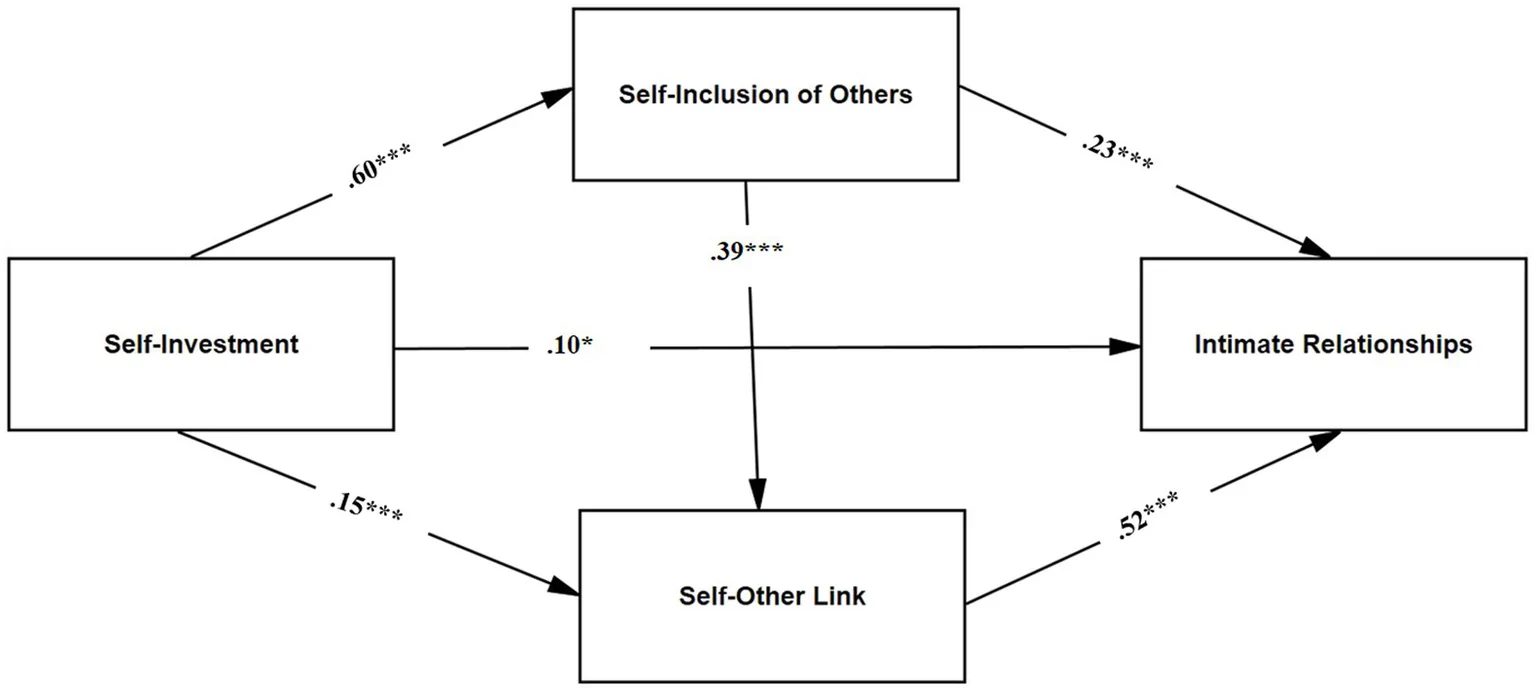
Plot of the model’s path coefficients. *p < 0.05; **p < 0.01; ***p < 0.001.

To obtain reliable results for the mediation test, a bias-corrected nonparametric percentile Bootstrap method was further used. A Bootstrap with a 95% confidence interval (CI) that does not include 0 indicates a significant mediating effect. As shown in Table 3, the direct effect value of self-investment on intimate relationships was 0.10, accounting for 22.73% of the total effect, with a 95% CI of [0.02, 0.18], indicating a significant direct effect. The total indirect effect value of self-inclusion of others and the self-other link in the impact of self-investment on intimate relationships was 0.34, accounting for 77.27% of the total effect, with a 95% CI of [0.27, 0.47], which confirms a significant mediating effect. This mediating effect consists of three indirect effect paths (see Table 3): The indirect effect of the path “self-investment → self-inclusion of others → intimate relationships” was 0.14, accounting for 31.82% of the total effect, with a 95% CI of [0.09, 0.19]; the indirect effect of the path “self-investment → self-other link → intimate relationships” was 0.08, accounting for 18% of the total effect, with a 95% CI of [0.03, 0.13]; and the chain mediating effect of the path “self-investment → self-inclusion of others → self-other link → intimate relationships” was 0.12, accounting for 27.27% of the total effect, with a 95% CI of [0.09, 0.16].

**Table 3:** Bootstrap analysis of significance tests for intermediate effects (*n* = 369).

Path	Effect	Effect size (%)	Boot LLCI	Boot ULCI
A → B → D	0.14	31.82%	0.09	0.19
A → C → D	0.08	18.00%	0.03	0.13
A → B → C → D	0.12	27.27%	0.09	0.16
Indirect effect	0.34	77.27%	0.27	0.47
Direct effect (A → D)	0.10	22.73%	0.02	0.18
Total effect	0.44	100%	0.34	0.54

A = Self-investment; B = Self-inclusion of others; C = Self-other link; D = Intimate relationships; Boot LLCI = Bootstrap Lower Limit Confidence Interval; Boot ULCI = Bootstrap Upper Limit Confidence Interval.

## 4 Discussion

### 4.1 The predictive role of self-investment

This study found that self-investment is associated with the self-inclusion of others and self-other link, and it can also predict intimate relationships either directly or indirectly. Thus, these findings indicate that self-investment may play a critical role in predicting these outcomes through multiple pathways. As mentioned, self-investment refers to the resources that individuals devote to interpersonal relationships, including time, emotion, and energy among others (Gerbasi and Prentice, 2013; Harandi, 2021). This present study highlights the self-expansion theory by proposing a process-oriented model in which self-investment may comprehend cognitive integration and has potential to proceed into emotional resonance (self-other link), which could shape intimate relationship quality through a sequential developmental process of these elements. Such investment can influence individuals’ cognition of others, emotional resonance, and the expansion of the self-concept, thereby affecting the quality of the self-inclusion of others, self-other link, and intimate relationships. Self-inclusion of others, again, refers to the degree to which individuals integrate others into their self-concept; the higher this degree, the more intimate the relationship between the two parties (Aron et al., 1991, 1992). The self-expansion model holds that people have a need to expand their self-concept, and intimate relationships are an important way to achieve this self-expansion (Ahn et al., 2024). However, in the sequential process, self-expansion theories have often been viewed as a static outcome of intimate relationship. Findings of this study demonstrate that self-investment may proceed cognitive and emotional phases of intimacy which lean toward the progressive developmental model. Existing studies have also found that linking relationships with self-expansion leads to increased relationship satisfaction; in this case, the relationship cognitively means incorporating the other person into the self (Montoya et al., 2018). By sharing experiences, resources, and identities with their partners, individuals can enrich their self-concept and increase self-complexity (Slotter and Gardner, 2009; Brown et al., 2009). When individuals invest more in the relationship, they are more likely to view their partners as part of “us” rather than just “you” and “me” (Weinstein et al., 2016). This demonstration of relational interdependence serves as a key motivator in Chinese collectivist cultures, as it can help reinforce the cultural layer of self-expansion processes rather than individual autonomy in relationships. This sense of “us” promotes mutual dependence and support between each other, further deepening the degree of self-inclusion of others. Empirical studies have shown that in romantic relationships, individuals tend to incorporate their partners’ characteristics, resources, and perspectives into their own cognitive structures, thereby increasing the degree of self-inclusion of others (Aron et al., 1992; Slotter and Gardner, 2009). This cognitive integration can help reduce the boundary between oneself and others and enhances intimacy and commitment in the relationship.

Self-other link refers to the degree to which individuals feel closely connected to others, and this sense of connection can affect individuals’ wellbeing and social behaviors (Zhang and Hommel, 2022; Galinsky et al., 2005). Self-investment can also enhance individuals’ ability of emotional resonance. When individuals invest more emotion in others, they are more likely to understand others’ feelings and needs, thereby promoting the self-other link (Zhang and Hommel, 2022). By actively listening to and understanding their partners’ perspectives, individuals can better think from the other party’s standpoint and reduce misunderstandings and conflicts (Galinsky et al., 2005). Perspective-taking also helps individuals recognize their common ground with others and strengthen the sense of connection between them. Cross-cultural studies have shown that cultural background can also affect the self-other link. For example, collectivist cultures emphasize interdependence between people, while individualistic cultures focus more on individual independence (Ng and Lai, 2009; Barakji et al., 2018). Therefore, in collectivist cultures self-investment may be more likely to promote the self-other link.

Intimate relationships refer to deep and long-lasting emotional connections established between individuals, which usually include romantic love, friendship, family relationships, among other close relationships (Felix et al., 2021; Shen, 2025). Self-investment is a key factor in maintaining the satisfaction of intimate relationships. When individuals invest sufficient time and energy in the relationship, their partners will feel valued and cared for, thereby improving relationship satisfaction (Shen, 2025). Individuals who invest in relationships are more inclined to adopt constructive conflict resolution methods. Additionally, they will be more willing to communicate, compromise, and seek joint solutions, rather than adopting destructive behaviors such as avoidance, aggression, or control (Harandi, 2021). Self-investment is closely related to long-term commitment. When individuals invest a lot of resources in the relationship, they are more likely to make efforts to maintain the relationship, thereby increasing the stability and durability of the relationship (Follingstad et al., 2012). The latest research shows that interpersonal mindfulness can improve satisfaction in intimate relationships (Pratscher et al., 2018). Interpersonal mindfulness refers to maintaining an attitude of focus, acceptance, and non-judgment in interpersonal interactions, which may also be a form of in-depth investment. By cultivating interpersonal mindfulness, individuals can better perceive their own and others’ emotions, thereby improving the quality of communication and emotional resonance.

### 4.2 The predictive role of self-inclusion of others

Self-inclusion of others plays a crucial role in understanding the connection between the self and others, as well as its relation to intimate relationships. Self-inclusion of others refers to the degree to which an individual integrates others into their self-concept. The model of this study emphasizes the cognitive role of IOS where it’s association with perceived emotional resonance of the self-other link helps support the process of a sequential development model of intimate relationships. A higher level of self-other overlap means that an individual cognitively perceives their partner as part of themselves, which can influence the individual’s behaviors and emotional experiences in intimate relationships (Quintard et al., 2020; Weinstein et al., 2016).

Theoretically, the self-inclusion of others exerts an impact on the self-other link, we also see this association within our model as well. The impact of this relationship, can be illustrated in a few ways, such as through emotional resonance and understanding. Individuals with a high level of self-inclusion of others are more able to empathize with their partners’ emotions and inclined to think from their partners’ perspectives, thereby promoting mutual understanding and support (Quintard et al., 2020). This emotional resonance helps establish deeper connections and overall enhance the intimacy of the relationship (Lin et al., 2019). Here, cognitive and emotional elements of intimacy interact dynamically which extends the theoretical comprehension of the step-by-step formation of intimacy. Another way in which self-inclusion of others and the association with the self-other link can be shown is from resource allocation and fairness. When individuals perceive their partners as part of themselves, they are more likely to allocate resources fairly and willing to face challenges together. This sense of commonality helps reduce conflicts and improve relationship satisfaction. Thirdly, motivation and subsequent behavior is another illustration. For instance, an individual may make more active efforts and be motivated to contribute to their partner’s happiness, as their partner’s success is also regarded as their own success, and thus they are more likely to change their behavior to achieve joint successes (Overall et al., 2010).

Findings of the present study suggest that self-inclusion of others may initiates the first cognitive steps of intimacy by reflecting on the partner’s identity; this occurs before deep emotional bonds surface through the self-other link. Furthermore, the associated role of self-inclusion of others in intimate relationships can be explained across literature in a few ways. With relationship satisfaction, for example, previous studies have noted its positive correlation between self-inclusion of others. The more an individual integrates their partner into their self-concept, the higher their relationship satisfaction (Shen, 2025). This may be because self-inclusion of others facilitates the development of emotional bonds, mutual support, and the achievement of shared goals. Self-inclusion of others also contributes to the maintenance of intimate relationships. When individuals experience a sense of fusion with their partners, they are more willing to invest time and energy in maintaining the relationship and coping with challenges, thereby reducing the risk of relationship breakdown. Self-inclusion of others can also predict the effectiveness of supportive behaviors. Individuals have a better understanding of their partners’ needs and can provide support that is more in line with their partners’ expectations, which in turn enhances their partners’ sense of wellbeing and relationship satisfaction (Quintard et al., 2020). Partner support is crucial for coping with life stress and maintaining relationship stability (Overall et al., 2010). Lastly, mutual support between partners contributes to each other’s self-improvement, which further enhances relationship quality (Overall et al., 2010). Supportive behaviors from partners, such as providing encouragement and assistance, can strengthen individuals’ self-confidence, sense of competence, and promote personal growth. The literature does explain there are a few reasons why these variables are connected; this study further supports this, finding significance between self-inclusion of others with both the self-other link and intimate relationships among the sample.

### 4.3 The predictive role of the self-other link

This study found that the self-other link has an associated role in intimate relationships, and this result can be explained by existing research. Our findings are homogenous with the existing theories that demonstrate that emotional resonance (self-other link) promotes intimacy; however, we extend this idea by positioning this within a sequential model—incorporating self-investment and cognitive inclusion. Existing studies have shown that the self-other link influences individuals’ satisfaction, sense of wellbeing, and behavioral patterns in intimate relationships.

Like our findings, literature has demonstrated a significant association between the self-other link and satisfaction in intimate relationships (Quintard et al., 2020; Hofmann et al., 2015). This can be explained as higher relationship satisfaction can promote individuals to form effective motivational mindsets for self-regulation, thereby facilitating the achievement and performance of goals (Hofmann et al., 2015). In romantic love, there is also an overlap in the psychological representations between the self and one’s partner; this overlap helps enhance the romantic experience and can predict relationship quality (Quintard et al., 2020). The higher the similarity in emotional responses those individuals perceive between themselves and their close ones, the higher their emotional wellbeing (Cheng and Grühn, 2016).

Additionally, this finding has been supported in literature examining how the self-other link affects individuals’ supportive behaviors and perceptions within relationships. Self-determination theory suggests that the overlap between the self and others is influenced by individuals’ self-determined motivation, which in turn affects partners’ perception of support and their sense of wellbeing (Weinstein et al., 2016). Individuals with low self-esteem, for example, tend to seek support in indirect ways, but this strategy often backfires, leading to rejection from close ones (Don et al., 2018).

Findings of our study suggest that emotional resonance may not be solely independent for emotional attachment; rather it may evolve through the sequential process of cognitive inclusion of the partner. Furthermore, literature examining attachment styles have been shown to also influence the self-other link and the quality of intimate relationships. A secure attachment style helps individuals exhibit a higher level of intimacy in friendships, including self-disclosure, responsiveness to others, and the perception of being understood and cared for (Grabill and Kerns, 2000). Attachment style can also explain why individuals’ and their friends’ perceptions of readiness for commitment affect the establishment and maintenance of relationships, where insecure attachments may impair such perceptions (Yang et al., 2025). However, it is important to note that for the present study, the observed relationships are correlational, given the cross-sectional nature of the study, and do not imply causality.

## 5 Research limitations and future implications

This study has limitations in its design and implementation. First, the study adopts a cross-sectional design, which can only capture the distribution and correlation of variables at a specific time point, failing to reveal the dynamic changes in the relationships between variables and making it difficult to establish the causal relationship among self-investment, intimate relationships, and mediating variables. This limits the causal explanatory power of the conclusions. Future studies can use a longitudinal prospective design to record the dynamic trajectories of variables through long-term follow-ups, combined with cross-sequential models or hierarchical linear models for analysis. They can also attempt experimental methods, manipulating independent and mediating variables and using pre-test/post-test comparison designs to verify causal mechanisms and improve the internal validity of conclusions. Second, although the study verifies the correlation between self-investment and intimate relationships as well as the role of mediating variables, it does not deeply explore the neurophysiological mechanisms behind this correlation. Subsequent studies can integrate technologies such as functional magnetic resonance imaging (fMRI) and event-related potentials (ERP), design multi-scenario experimental tasks, observe brain region activation and neural responses, and reveal the relevant neural mechanisms. This will provide more solid physiological evidence for the conclusions and expand an interdisciplinary perspective for understanding the nature of human intimate relationships and the development of self-cognition. Third, this present study recruited a majority of (74%) Chinese female participants through convenience sampling technique, which may limit the generalizability of the findings. Future longitudinal studies should focus of the replication of the findings in more diversified samples, including different demographic, cultural and gender groups to comprehend the broader applicability of those associations. Fourth, although Harman’s single-factor test was employed to assess CMV, it has some limited sensitivity for detecting subtle forms of bias in self-report data. Future studies could be benefitted from replicating the findings using multi-method for data collection.

## 6 Conclusion

In summary, this study provides a distinctive exploration of the relationship between self-investment and intimate relationships among Chinese college students. The results bring to light the mechanisms through which self-investment, self-inclusion of others, and the self-other link affect intimate relationships; thereby enriching the empirical evidence in this field and within a specific cultural context. The research found that self-investment significantly predicts intimate relationships, with self-inclusion of others and self-other link serving as chain mediators. This suggests that building high-quality intimate relationships can be a comprehensive process involving multiple interactive pathways, with both direct and proactive emotional expression, as well as indirect and subtle mutual integration. Therefore, on a practical level, individuals should be encouraged to actively engage in behaviors and activities that promote deep integration between oneself and others, while strengthening interpersonal bonds through genuine self-disclosure and proactive mutual exploration—as it thus is associated with establishing and sustaining high-quality intimate relationships.

## Data Availability

The original contributions presented in the study are included in the article/supplementary material, further inquiries can be directed to the corresponding author.
